# Intestinal aging-related immune dysfunction: mechanisms and interventions: Intestinal aging-related immune dysfunction

**DOI:** 10.3724/abbs.2025157

**Published:** 2025-09-05

**Authors:** Xin Shen, Xianzhi Gao, Lie Wang

**Affiliations:** 1 Co-Facility Center Zhejiang University School of Medicine Hangzhou 310058 China; 2 Zhejiang University School of Medicine Hangzhou 310058 China; 3 Liangzhu Laboratory Zhejiang University Medical Center Hangzhou 311121 China; 4 Institute of Immunology and Bone Marrow Transplantation Center First Affiliated Hospital Zhejiang University School of Medicine Hangzhou 310024 China

**Keywords:** intestinal immunosenescence, inflammation, gut microbiota, intestinal barrier, age-related diseases

## Abstract

Intestinal immunosenescence, a hallmark of organismal aging, has emerged as a critical biological process impacting the health of elderly individuals. This review systematically examines the core mechanisms underlying intestinal immunosenescence, including immune cell dysfunction, imbalances in immune-microbiota interactions, and impaired barrier function. We analyze its associations with infectious diseases, chronic inflammation, and neurodegenerative disorders, summarizing recent advances in dietary interventions, microecological therapy, and other emerging strategies. By integrating cutting-edge technologies, we prospect the development of precision interventions aimed at delaying intestinal immunosenescence, thereby providing a theoretical basis for improving the healthspan of the aging population.

## Introduction

The intestine serves as the largest immune organ in the human body, harboring approximately 70%–80% of immune cells and constituting the first line of defense against pathogens [ 1, 2] . Its unique immune architecture includes Peyer’s patches (PPs), intraepithelial lymphocytes (IELs), lamina propria lymphoid tissues, lymphoid follicles and mesenteric lymph nodes (MLNs), which form a multilayered defense system that plays a key role in maintaining immune balance [3]. Intestinal immunity exhibits dual functions. On the one hand, it recognizes pathogen-associated molecular patterns (PAMPs) through pattern recognition receptors (PRRs) to activate innate immune responses and eliminate invading microbes [ 4– 6] . On the other hand, it develops immune tolerance to the commensal microbiota by regulating immune cell subsets such as T cells and innate lymphoid cells (ILCs) [ 7, 8] , thus maintaining the host-microbiota symbiotic relationship. Disruption of this dynamic balance is closely related to various diseases, and the decline in gut immune function during aging further exacerbates this imbalance.

Immunosenescence refers to the gradual decline in immune system structure and function with aging, ultimately leading to poor vaccination efficacy, persistent low-grade inflammation, increased infection susceptibility, and age-related disease onset [ 9– 11] . In the intestinal microenvironment, aging exerts detrimental effects on the mucosal immune system by impacting immune cells, cytokines, and signaling pathways, thereby disrupting the intestinal immune barrier and leading to immune dysfunction. This disruption can subsequently trigger a variety of immune-related diseases. In addition to the immune barrier, the intestinal barrier also comprises biochemical, physical, and biological barriers, which collectively coordinate the transport of ions and nutrients, immune regulation, and the intricate balance of gut microbiota diversity [12]. In the aging process, the expression of tight junction proteins decreases, and the mucus layer thins, leading to increased intestinal permeability [ 13, 14] . This destruction of the physical barrier alters the microenvironment of bacterial growth, thereby affecting the composition and function of the gut microbiota [ 15, 16] . In addition, aging-induced changes in the gut microbiota can also compromise the integrity of the intestinal mucosa and gut homeostasis, subsequently leading to diminished immune responsiveness and regulatory capacity and an inability to effectively counteract various exogenous insults [17]. The decline in immune system function, in turn, also impacts the gut microbiota, resulting in a sustained decline in immunity [18].

With the World Health Organization predicting 1.6 billion people aged ≥ 65 worldwide by 2050 [19], the incidence of aging-related diseases ( *e*. *g*., infectious diseases, inflammatory bowel disease, Alzheimer’s disease) is expected to rise significantly. As a core hub of the immune-metabolic-neural axis, intestinal immunosenescence not only impacts local defense but also regulates systemic health via the gut-brain, gut-lung and gut-liver axes [ 20, 21] . Given the critical role of the intestinal immune system in health and disease, understanding the mechanisms underlying immunosenescence and its impact on intestinal immunity is essential for developing interventions to promote healthy aging. Recent studies have highlighted the potential of modulating the gut microbiota through dietary interventions, microecological therapy, and fecal microbiota transplantation (FMT) to restore immune function and reduce inflammation [ 18, 22– 24] . These approaches offer promising avenues for mitigating the effects of immunosenescence and improving the quality of life of elderly individuals.

This review aims to explore the key aspects of intestinal immunosenescence, including its mechanisms, the role of the gut microbiota in immune regulation, and the implications for age-related diseases. We also discuss the current research status of interventions that may help reverse or mitigate the effects of immunosenescence in the gut. By integrating the latest findings from both basic and clinical studies, we provide a comprehensive overview of the challenges and opportunities in the field of gut immunity and aging.

## Mechanisms of Intestinal Immunosenescence

The gut is a central organ in the human body that serves as the primary interface between the external environment and the internal immune system. The intestinal mucosal immune system, which includes gut-associated lymphoid tissues (GALTs), is indispensable for maintaining immune homeostasis and defending against pathogens. It has been demonstrated that intestinal mucosal immune function decreases with age [25]. The gut microbiota, a complex community of microorganisms residing in the gastrointestinal tract, influences both innate and adaptive immune responses. With age, the composition and diversity of the gut microbiota undergo significant changes, often leading to a reduction in beneficial bacteria and an increase in pathogenic species [16]. This microbial imbalance can disrupt the gut barrier, promote systemic inflammation, and impair immune function. The intestinal barrier, which is composed of the luminal microbiota, the mucus layer, immune cells, and the physical barrier, which consists of epithelial cells, plays a crucial role in preventing bacterial toxins and pathogens from entering the intestinal lumen into the circulation [26]. However, aging damages the integrity of the intestinal barrier and its regulatory function [27]. An impaired barrier function or even minor changes in the regulation of the epithelial or biochemical barrier may lead to chronic immune activation, contributing to local and systemic diseases, including coeliac disease. In summary, the interplay among immune homeostasis, microbial balance, and barrier function is intricate and significantly impacts intestinal immunosenescence.

### Decline in immune cell function

#### Paracrine effects of senescence-associated secretory phenotype (SASP)

**Figure 1:**
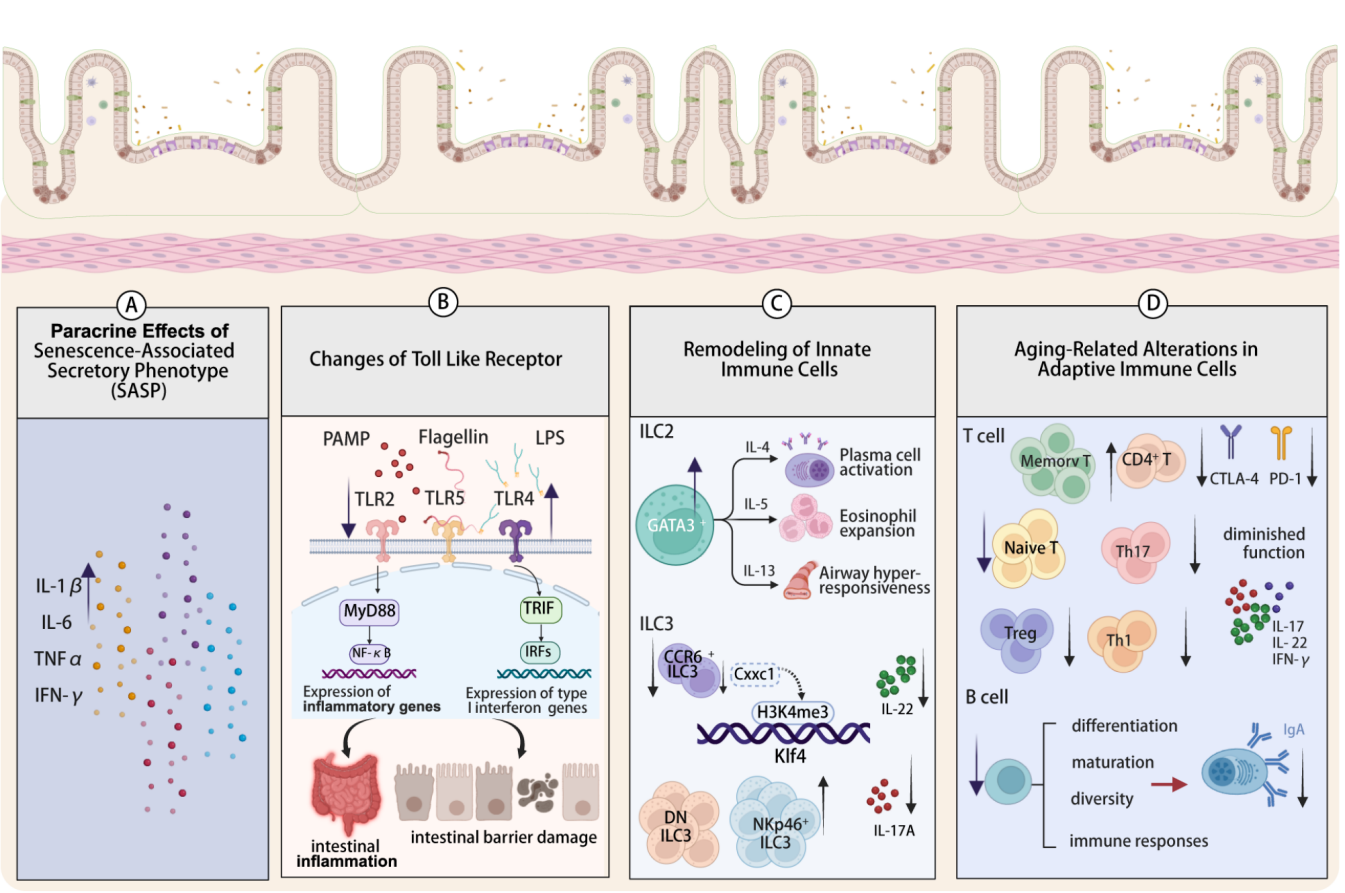
Figure 1 Age-related changes in intestinal immunity During senescence, the intestine gains immune changes in terms of the SASP, TLRs, and innate and adaptive immune cells. These changes in the aged intestine are responsible for many overall age-related diseases, such as infectious diseases, chronic inflammation, and neurodegenerative disorders. Created in BioRender. An J (2025) https://BioRender.com/33ukol8.

As organisms age, a secretory phenotype known as the SASP develops, which results in the production of high levels of inflammatory bioactive factors that can impact surrounding tissues and promote disease [28]. In the intestine, the levels of pro-inflammatory cytokines, particularly tumor necrosis factor α (TNF-α), interferon-γ (IFN-γ), interleukin (IL)-1β, and IL-6, tend to increase with increasing age [ 17, 27] , which can lead to the formation of a pro-inflammatory microenvironment ( Figure 1A). Inflammatory pathways such as the p38-MAPK, JAK-STAT, and NF-κB pathways are activated by these inflammatory factors, resulting in intestinal epithelial cell damage and cellular senescence [29]. Inflammatory bowel disease (IBD), including Crohn’s disease (CD) and ulcerative colitis (UC), is a recurrent and incurable inflammatory disorder of the gastrointestinal tract. In the IBD intestine, IL-6 and IL-1β can upregulate the expression of CDKN1A and CDKN2A through the JAK-STAT signaling pathway, leading to cell cycle arrest and inducing stem cell senescence [ 30, 31] . TNF-α not only directly induces the DNA damage response (DDR) and p53 activation but also accelerates telomere shortening and cell senescence [ 32, 33] . Additionally, SASP factors promote M1 macrophage polarization and amplify inflammation while suppressing the anti-inflammatory function of M2 macrophages, preventing the resolution of inflammation [34]. This persistent inflammatory state disrupts the delicate balance of immune responses within the gastrointestinal tract.

#### Remodelling of innate immune cells

##### Abnormal TLR signaling and anti-inflammatory-pro-inflammatory imbalance

Toll-like receptors (TLRs), a type of PRR, respond to specific microbial ligands and danger signals produced by the host during infection and initiate downstream cascades that activate both innate and adaptive immunity [35]. TLRs are expressed on the plasma membranes and endosomes of immune cells such as macrophages and dendritic cells (DCs). Aging leads to abnormal expression of multiple TLRs and impairment of the intestinal barrier, thereby enhancing intestinal susceptibility [36]. Among these, TLR2, TLR4, and TLR5 play crucial roles in the innate immunity of the intestinal mucosa [ 37, 38] ( Figure 1B). TLR2 is closely related to the integrity of the epithelial barrier. With increasing age, the expression and function of TLR2 in mice are significantly reduced, which affects the levels of IL-10 and IFN-γ and consequently leads to intestinal mucosal damage and colitis [39]. Additionally, the level of trefoil factor 3 (TFF3), an important colonic protective and repair factor, decreases over time in mice and is negatively regulated by TLR2 signaling [39]. TLR4 can be activated by specific exogenous substances, such as endotoxins and lipopolysaccharides (LPS), thereby triggering an innate immune response and inflammation through MyD88-dependent and TRIF-dependent signaling pathways [40]. Overactivation of TLR4 results in the production of pro-inflammatory cytokines, including IL-1, IL-6, and IL-8, while simultaneously suppressing the expression of anti-inflammatory factors. This disruption of immune balance can trigger chronic inflammatory responses [ 38, 41] . The expression levels of TLR4 in the colonic contents and mucosa of aged mice are significantly elevated, which parallels multiple signs of low-grade intestinal inflammation [41]. Kim *et al*. [42] reported that TLR4 is directly activated by the LPS fraction of the gut microbiota of aged mice, leading to an increased inflammatory response in macrophages. These age-related changes in TLR2/4 expression and function, combined with alterations in cytokine levels and mucosal repair mechanisms, collectively contribute to increased susceptibility to intestinal inflammation in elderly individuals. TLR5 maintains the balance of the gut immune system by interacting with *Escherichia coli* and preventing diseases arising from disruptions in microbial equilibrium [43]. Although TLR5 expression is relatively stable in aged mice and older individuals [44], a recent study has shown that activation of TLR5 via nasal administration of FPs (a flagellin-containing fusion protein) in aged mice effectively regulates the intestinal mucosal immune system, resulting in increased production of IgA and IL-22 and retention of the young intestinal structure [45]. This study demonstrated that mucosal activation of TLR5 can extend lifespan and reduce age-related health defects.

Aging is also associated with alterations in DCs and macrophages. DCs, which perform powerful functions such as antigen presentation and T-cell activation, play pivotal roles in the immune response process [ 46, 47] . DCs sense and capture pathogens via the expression of PRRs, such as TLRs. Panda *et al*. [48] demonstrated a statistically significant, age-associated decrease in the expression of TLR3 and TLR8 in myeloid DCs (mDCs) and of TLR7 in plasmacytoid DCs (PDCs). There is no major effect on the numbers or phenotypes of DC subsets in aged subjects, except that the number of pDCs in the circulation has been observed to decrease [49]. Aging reduces the tolerance of DCs, resulting in functional changes such as insufficient migration and decreased production of specific cytokines, both of which are necessary to stimulate protective T-cell responses [ 50, 51] . In elderly individuals, DCs secrete higher levels of pro-inflammatory cytokines while producing lower levels of anti-inflammatory and immune-regulatory cytokines, fostering chronic inflammation during aging. Additionally, aged DCs display heightened immune responses against self-antigens, further exacerbating inflammation and undermining tolerance [49]. Such cumulative immune deficits could collectively account for the blunted and often ineffective host defenses observed in elderly populations.

Macrophages possess robust phagocytic capacity that clears damaged or senescent cells and dampens inflammation. However, aging-induced gene mutations and chemical exposure drive macrophage senescence, which manifests as a special form of durable cell cycle arrest and chronic low-grade inflammation, such as SASP [34]. Persistent senescence of macrophages may trigger a series of severe consequences, such as a reduction in their phagocytic function, the rewiring of autophagy and metabolic signaling, ultimately resulting in impaired bacterial clearance and elevated pro-inflammatory cytokines, while the immune balance of recombinant macrophages may reverse aging [ 52– 55] . Compared with their young counterparts, when macrophages co-cultured with CD4 ^+^ T cells, aged macrophages exhibit reduced suppression of lymphocyte proliferation, which indicates that cell-intrinsic factors and the aged microenvironment drive an age-dependent loss of the anti-inflammatory phenotype [56]. Moreover, diminished expression of the longevity gene FoxO3 is observed in aged macrophages that have lost their anti-inflammatory behavior relative to those from young mice. Moreover, TLR expression on macrophages from elderly individuals is markedly reduced, which may compromise T-cell function and precipitate broader immune defects [34].

##### Epigenetic regulation of ILC3 subset dysregulation

ILCs are innate counterparts of T cells that lack diverse antigen receptors but contribute to tissue immunity, homeostasis, and inflammation [57]. On the basis of their transcription factor expression and cytokine production, ILCs can be further classified into three groups. Among them, group 2 ILCs (ILC2s) express GATA binding protein 3 (GATA3) and secrete IL-4, IL-5, and IL-13 to promote host resistance to helminth infection. Souza *et al*. [58] reported that the number of ILC2s in the small intestinal lamina propria (SILP) of aged mice greatly increased with aging [58] ( Figure 1C). ILC3s, which phenotypically mirror T helper 17 (Th17) cells, are pivotal for intestinal mucosal immunity and secrete IL-22 and IL-17A to maintain the epithelial barrier and antimicrobial defense. ILC3s can be classified into three heterogeneous categories according to the expression of the chemokine receptor CCR6 and natural cytotoxicity-triggering receptor (NCR): CCR6 ^+^ ILC3s, NKp46 ^+^ ILC3s and CCR6 ^-^NKp46 ^-^ double-negative (DN) ILC3s. Our previous research confirmed that aged mice exhibit aberrant ILC3 cell subset proportions and functions, leading to increased susceptibility to bacterial and fungal infection [59] ( Figure 1C). Mechanistically, this imbalance is mediated by the epigenetic regulator Cxxc1-H3K4me3-Klf4 axis. Age-related downregulation of Cxxc1 results in decreased H3K4me3 modification, which in turn inhibits Klf4 binding and reduces the differentiation of CCR6 ^+^ ILC3s. Overexpression of Klf4 in aged mice restored CCR6 ^+^ ILC3 proportions, increasing IL-22 and IL-17A secretion. Rizk *et al*. [60] underscored the importance of the cellular inhibitor of apoptosis proteins 1 and 2 (cIAP1/2) in sustaining ILC3s during the aging process, thereby promoting effective barrier immunity. Another article reported that in aged human donors, ILC3s are decreased in the intestine compared with those in young donors [61].

#### Aging-related alterations in adaptive immune cells

##### Thymic involution and T-cell dysregulation drive gut mucosal immunosenescence

One of the hallmarks of immunosenescence is the involution of the thymus, the major organ responsible for the generation of a highly diverse but selected T-cell repertoire [ 62– 64] . Age-associated thymic involution leads to diminished generation of naive T cells that reach the periphery, a reduction in the diversity of the peripheral T-cell repertoire and compensatory clonal expansion of memory T cells, causing T-cell aging and compromising the detection of pathogens ( Figure 1D). Aged T cells exhibit characteristics of inflammatory responses and unhealthy aging, such as mitochondrial dysfunction, epigenetic remodeling, DNA damage and short signs of short telomeres, which activate aging-related signaling pathways [65]. Senda *et al*. [66] reported an age-related decrease in naive T cells in the GALT, as well as in the lymph nodes and spleen. Aging impairs the proliferation of CD4 ^+^ T cells in PPs. The underlying mechanism may be that aging alters the responsiveness of CD4 ^+^ T cells to T-cell growth factors and diminishes the synthesis of cytokines by CD4 ^+^ T cells [67]. The immune checkpoint molecules programmed death receptor 1 (PD-1) and cytotoxic T-lymphocyte antigen 4 (CTLA-4) reduce the number of CD4 ^+^ T cells in the intestinal lamina propria (LP) with aging [ 68, 69] . As coinhibitory molecules, CTLA-4 and PD-1 can inhibit T-cell activation and T-cell responses through distinct mechanisms, which may account for CD4 ^+^ T-cell hyperactivation as well as the mucosal inflammatory environment in aging individuals [70]. The frequency and function of Th17 and Th1 cells derived from elderly individuals are impaired, including their responsiveness to the gut microbiota and proliferative capacity following antigen exposure [68]. Given that a lack of intestinal Th17 cells is associated with epithelial barrier damage and microbial translocation, it is likely that defects in Th17 and Th1 cells in elderly individuals play a significant role in the aging of the gut mucosal immune system [25]. With increasing age, the ratio of effector cells to regulatory T cells (Tregs) in mice changes, which may in turn lead to a decline in immune response capability [ 65, 71] . DCs are the sentinels of the immune system that maintain immune tolerance through cytokine and Treg generation. The gradual, age-related erosion of DC tolerance compromises their capacity to optimally drive Treg generation. A reduction in the number of Tregs further impairs the tolerance of the immune system, leading to excessive or dysregulated immune responses and the formation of a vicious cycle [51].

##### B-cell differentiation impairment and reduced IgA secretion

Intes-ti-nal B cells, which are located primarily in the LP and GALT, differentiate into plasma cells to secrete immunoglobulin A (sIgA) [72]. In aged mice, the capacity for B-cell differentiation and maturation is diminished, which in turn leads to a reduction in B-cell diversity and alters the distribution of peripheral B cells ( Figure 1D). This impairment affects B-cell functions, resulting in a significant reduction in immune responses and a marked decrease in antibody production. Additionally, B cells from elderly individuals produce relatively high levels of TNF-α, which further disrupts intestinal immune homeostasis [73]. sIgA serves as a primary line of defense against potentially invasive pathogenic microorganisms and participates in host-microbe interactions by shaping the composition of the gut microbiota and maintaining intestinal homeostasis [74]. Aging impairs the migration of IgA immunoblasts to the intestinal lamina propria and reduces antibody titers, leading to a diminished mucosal immune response [75]. This decline in homing capacity is linked to the reduced expression of adhesion molecules such as α4β7, which are essential for the directed migration of immune cells [76]. As a result, elderly individuals are more susceptible to bacterial and viral gastrointestinal infections. Over the years, the intestine has undergone immune degeneration, which exacerbates systemic aging.

### Bidirectional interactions between the gut microbiota and immunosenescence

#### Core microbiota imbalance and functional decline

The gut microbiota, often referred to as a “hidden endocrine organ”, plays a key role in maintaining host homeostasis by regulating metabolism, immunity, and aging processes [28]. The gut microbiota is dominated by bacteria but also includes fungi, viruses, and archaea. Bacteria from the phyla *Firmicutes* (associated with better health phenotypes) and *Bacteroidetes* are the most prevalent, constituting approximately 80%–90% of the total microbiota [ 77, 78] . Owing to the influence of several environmental and genetic factors, the species and genera of the gut microbiota in individuals are significantly different. The abundance of core commensal bacteria decreases, and at the same time, the abundance of opportunistic and pathogenic microorganisms increases [79], indicating that the aged intestine is not protected from commensal bacteria. It can impair the intestinal mucosal barrier, increase the risk of intestinal infections, trigger chronic intestinal inflammation, and exacerbate the process of aging [ 17, 25, 80, 81] . For example, the composition of the gut microbiota becomes imbalanced with age, with a significant decrease in the ratio of *Firmicutes* to *Bacteroidetes* (10.9 in young adults and 0.6 in elderly individuals) [82]. The abundance of *Bifidobacteria*, which are generally believed to benefit the gut health by inhibiting the growth of harmful bacteria and resisting pathogen infections, is decreased in elderly individuals. Compared with younger adults, the majority of adults over 65 years of age present reduced microbiota diversity [83]. In addition, aging causes intrinsic functional degradation of the gut microbiota, such as evolution and mutation. Moreover, variations in the composition and function of the gut microbiota significantly influence the modulation of the production of cytokines, particularly TNF-α and IFN-γ.

Centenarians, who are individuals over the age of 100, are considered the most successful biological aging model in humans. The common characteristics of this population include a low prevalence of chronic diseases and well-maintained functionality and independence, thus determining a healthy phenotype of successful aging. Luan *et al*. [84] reported that the Chinese Hainan Centenarian Cohort ( *n* = 146, aged 96–113 years) was dominated by *Bacteroides* and *Escherichia*. A more recent study on centenarians from Guangxi, China, ( *n* = 297, aged 100–117 years) has shown that centenarians exhibit gut microbiota features associated with younger individuals, characterized by an overrepresentation of a *Bacteroides*-dominant enterotype, increased species evenness, enrichment of bacteria with beneficial potential, and a reduction in potential pathobionts [85]. A comparison of fecal samples from different time periods revealed that with increasing age, the gut microbiota composition of centenarians becomes more stable, and the interindividual differences among centenarians decrease regardless of their health status. However, there are also conflicting findings regarding the microbial signatures of centenarians. Biagi *et al*. [86] conducted studies on Italian centenarians (aged 99–104 years) and reported microbiota similarities between young adults and seventy-year-olds but significant differences in centenarians. In centenarians, an increase in facultative anaerobes (opportunistic pro-inflammatory bacteria) was found to be closely related to the plasma levels of IL-6 and IL-8. Another study in Italy revealed that semisupercentenarians ( *n* = 24, aged 105–109 years) were specifically enriched in the genera *Akkermansia*, *Bifidobacterium*, and *Christensenella* [87]. Differences in the gut microbiota have been observed between centenarians in rehabilitation hospitals and those living at home. Compared with their community-dwelling counterparts, centenarians in rehabilitation facilities present higher levels of *Bacteroidetes*, lower bacterial diversity, and lower *Faecalibacterium* abundance [88]. These discrepancies may arise from variations in study populations, analytical methods, and environmental factors, highlighting the need for further research to clarify the causal relationships and specific mechanisms underlying the changes in the gut microbiota of centenarians and healthy longevity.

#### Roles of immunomodulatory microbiota metabolites

Moreover, the gut microbiota can affect intestinal mucosal immune function through its metabolism. Short-chain fatty acids (SCFAs), including butyrate, acetate, and propionate, are generated by bacterial fermentation of dietary fibers and resistant starch. Aging is associated with a decline in saccharolytic potential, reduced colonic lactate utilization, increased creatine catabolism, and decreased production of cobalamin, biotin and SCFAs [25]. SCFAs, especially butyrate, in the gut of elderly individuals are significantly reduced, which is closely related to the decreased abundance of SCFA-producing bacteria, such as the genera *Clostridium*, *Firmicutes*, *Bifidobacteria*, and *Lactobacillus* [89]. Additionally, the bile acid pool composition is affected [ 90, 91] . These microbial metabolites are crucial in modulating immune responses. For example, SCFAs can inhibit NF-kB and TNF-α, stimulate IL-8 secretion [92], promote neutrophil recruitment, induce Treg differentiation [93], enhance regulatory B-cell differentiation, and inhibit the differentiation of plasma cells [94] to regulate systemic immune responses. Consequently, SCFAs have an anti-inflammatory effect, and a reduction in SCFAs can increase intestinal inflammation. Butyrate enhances memory responses in CD8 ^+^ T cells and regulates immune tolerance by inducing Tregs and IL-10-producing T cells while inhibiting pro-inflammatory cytokines [95]. Bile acids can inhibit the production of pro-inflammatory cytokines and chemokines in monocytes and macrophages [96], whereas secondary bile acids, such as lithocholic acid, regulate immune responses by inhibiting Th17 cells and promoting Tregs [97]. Additionally, gram-negative bacteria produce lipopolysaccharides (LPS), which can induce receptor expression and activate relevant immune cells at appropriate levels. However, with aging, the release of LPS increases [42], and the accumulated LPS can activate TLRs, leading to decreased expression of tight junction proteins in the gut and inducing the development of systemic low-grade inflammation. LPS can also inhibit the Hedgehog signaling pathway in the gut via TLR2, weakening gut barrier function [98]. However, the gut microbiota of centenarians has stronger functions in SCFA metabolism, amino acid metabolism, and lipoic acid metabolism than their younger offspring do [99].

Tryptophan is one of the essential amino acids in humans and plays a crucial role in maintaining the balance of the gut microbiota and immune tolerance. In the metabolism of the gut microbiota, tryptophan metabolites include mainly indole, indole acid derivatives, tryptamine, *etc*. [100]. Among the bacterial metabolites of tryptophan, indole, a major molecule, is regarded as a beneficial signal in intestinal epithelial cells. It is produced mainly by intestinal bacteria such as *Escherichia coli*, *Proteus*, and *Bacteroides*. Indole activates the aryl hydrocarbon receptor (AhR) signalling pathway and promotes the production of IL-22. AhR facilitates the generation of Tregs in the gut and mediates IL-17 responses, thereby contributing to the improvement of intestinal inflammation and the maintenance of gut homeostasis [101]. Additionally, the AhR signaling pathway enhances the mucosal barrier and mucin production by inducing the expression of related genes, further consolidating the intestinal barrier. However, with increasing age, tryptophan and its derivative indole significantly decrease [102]. The immune functions of indole through AhR signalling and IL-22 become dysregulated, leading to epithelial barrier disruption and the occurrence of age-related diseases [103].

The gut microbiota plays a pivotal role in polyamine production. Polyamines have been implicated in adaptive immune system functions, including B-cell lymphopoiesis and activation as well as T-cell activation [104]. However, specialized immunosuppressive cell populations within the tumor microenvironment require high polyamine levels to sustain their growth and metabolism. Spermidine, the most common polyamine with important physiological functions, exerts anti-aging and immunomodulatory effects by regulating autophagy and histone deacetylation [105]. Intestinal bacteria such as *Bifidobacteria* (LKM512) can synthesize spermidine, and supplementation with this strain can increase intestinal and fecal spermidine levels, thereby potentially benefiting gut health [ 106, 107] . However, the abundance of *Bifidobacteria* decreases with aging, which may compromise the immune function of polyamines. Moreover, polyamine levels decrease with age across multiple organisms [ 105, 108] . Intriguingly, healthy nonagenarians and centenarians retain relatively high spermidine levels [109].

#### Immunosenescence weakens the antibacterial barrier

The mucosal immune system sculpts the gut microbiota by permitting commensal bacteria to colonize mucosal ecological niches while selectively eradicating or neutralizing harmful microorganisms. Intestinal immunosenescence not only directly impacts the recognition and processing of the gut microbiota [ 110, 111] but also promotes intestinal microbial dysbiosis via other mechanisms. For instance, the expression of proteins with immune functions, including antimicrobial peptides (AMPs) and IgA, changes with age, affecting the balance of the microbiota and increasing susceptibility to bacterial or viral infections in elderly individuals. Human α-defensin 5 (HD5), an AMP, is expressed at lower levels in elderly individuals than in middle-aged individuals. This reduction in HD5 expression compromises the intestinal immune system, changes the microbial composition and increases the risk of certain age-related diseases [112]. In addition, the IgA response to *Clostridiaceae* and *Enterobacteriaceae* diminishes with increasing age [113].

The gut microbiota plays a significant role in immune system regulation, and the delicate balance of intestinal immune homeostasis also affects the gut microbiota. With increasing age, the gut microbiota and its metabolites, as well as intestinal immune function, undergo corresponding changes in elderly individuals. The interaction between the gut microbiota and immunosenescence should not be underestimated.

### Multidimensional impairment of intestinal barrier function

**Figure 2:**
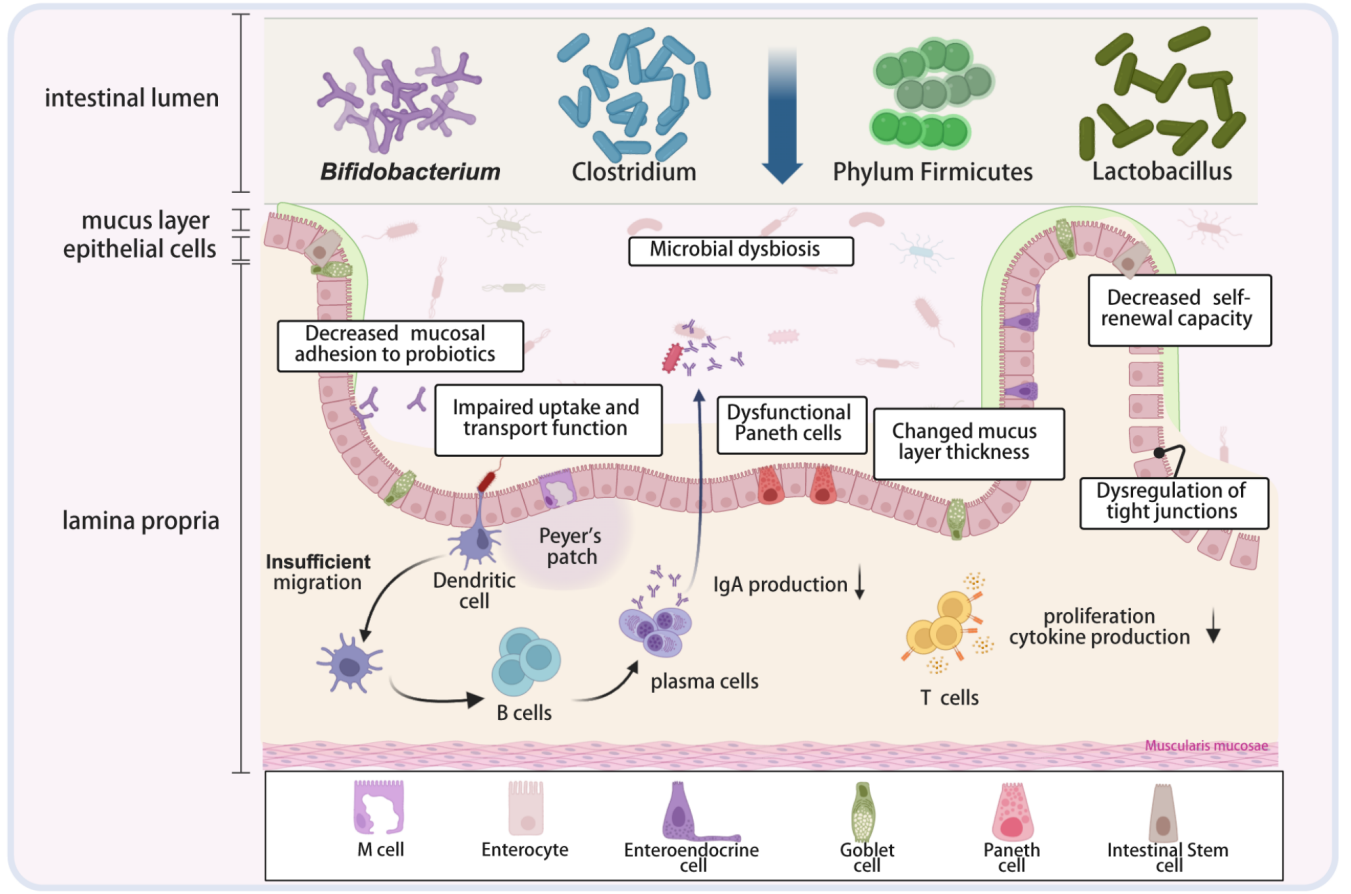
Figure 2 The impact of aging on the intestinal barrier The intestinal barrier includes microbial, biochemical, physical, and immunological barriers. As the host ages, a multitude of changes occur, leading to intestinal barrier and mucosal immune dysfunction. Created in BioRender. An J (2025) https://BioRender.com/33ukol8.

The gut serves as a vital barrier against external threats, such as microbes and toxins, and is a primary tissue for the interaction between organisms and the environment. The relationship between intestinal barrier dysfunction and aging has long been recognized ( Figure 2). Age-associated intestinal barrier dysfunction can lead to a variety of diseases in both animals and humans, most notably IBD, as well as chronic heart failure and asthma [ 114– 116] . Microbial, biochemical, physical and immune barriers together constitute the intestinal barrier. The gut microbiota and the immune system closely interact, which is crucial for maintaining the normal function and balance of the immune system. The gut microbiota maintains the integrity of the epithelial barrier and shapes the mucosal immune system, balancing host immune defense and oral tolerance through microbial metabolites, components, and attachment to host cells. To avoid abnormal immune responses, intestinal epithelial cells (IECs) isolate the gut microbiota from immune cells by constructing biochemical and physical barriers, thereby establishing a symbiotic and mutually beneficial relationship with the host. The physical barrier consists of the epithelial monolayer, which includes enterocytes, goblet cells, enteroendocrine cells, Paneth cells, and microfold cells (M cells) [26]. All of these factors contribute to the complex interplay of nutrient absorption while maintaining the mucosal barrier to prevent the permeation of bacterial toxins or pathogens and the secretion of immunological mediators. The biochemical barrier is composed of mucus and AMPs, with the former consisting primarily of Muc2, a highly O-glycosylated protein produced by goblet cells, and the latter being evolutionarily conserved, natural antibiotics produced by immune and epithelial cells in the gut. Within the lamina propria, various immune cells, such as macrophages, DCs, ILCs, T cells, B cells and mast cells, interact with the intestinal barrier.

#### Tight-junction disruption and stem-cell exhaustion: dual collapse of the aged mechanical barrier

As the core of the intestinal barrier, the intestinal epithelial layer prevents access to macromolecules and microbes while allowing a continuous influx of water, ions and nutrients. IECs are tightly connected by junctional complexes, which consist of tight junctions, adherence junctions and desmosomes on the lateral side of the basement [26]. Tight junctions are the key to maintaining intestinal permeability, a vital functional characteristic of the intestinal epithelial barrier. Aging is associated with the loss of tight junction proteins in the intestine. Hollander *et al*.[ 117, 118] reported that aged rats exhibited increased permeability to macromolecules, with permeability further increasing with age. Studies in non-human primates, such as aged baboons, have shown reduced expression of critical tight junction proteins such as Zona Occludens 1 (ZO-1), occludin and junctional adhesion molecule-A (JAMA-1), leading to increased permeability for horseradish peroxidase (HRP) [119]. In contrast, the expression of ZO-1, occludin and JAMA-1 is not altered in healthy elderly individuals (67–77 years old), and overall permeability to macromolecules is not affected compared with that in younger controls (20–40 or 7–12 years old) [120]. Only claudin-2, a pore-forming protein that increases permeability, is upregulated in the aged human gut. This enhances intestinal permeability to pathogens and toxins, which is associated with chronic low-grade inflammation and higher risk of intestinal infections in elderly individuals [25]. In addition, in patients with irritable bowel syndrome (IBS), the intestinal permeability of elderly individuals is greater than that of young individuals [121].

All subtypes of IECs are derived from pluripotent intestinal stem cells (ISCs) that reside in intestinal crypts. Given that the growth of intestinal organoid-derived aged mice is reduced compared with that of their younger counterparts, the proliferation and self-renewal ability of ISCs are greatly diminished with increasing age. In addition to their decline in growth, the ISCs of aged mice also highly express proapoptotic genes, indicating that the survival rate of these cells decreases [122]. As a result, the ability to recover from experimentally induced intestinal injury also decreases [123]. Other alterations in ISCs, including chronic activation and misdifferentiation, may also be critical in the development of intestinal barrier dysfunction during aging [ 27, 124] . Canonical Wnt proteins, which play a key role in regulating pleiotropic cell functions, including self-renewal, proliferation, differentiation and exercise, are decreased in ISCs, Paneth cells, and subepithelial mesenchymal cells in aged mice compared with young mice [125]. The production of Notum, a Wnt inhibitor that impairs regeneration of the aged intestinal epithelium, is increased in Paneth cells of aged mice [126]. M cells are specialized epithelial cells in the small intestine that allow antigen-presenting cells to transport antigens (especially particulate antigens) from the intestinal lumen to PPs and are crucial for inducing the production of IgA. In aged mice, mature M-cell density in the follicle-associated epithelium of PPs is dramatically reduced, impairing antigen-presenting function [127]. Moreover, in aged mice, IgA is significantly decreased, T-cell proliferation is reduced, and antigen-specific T-cell cytokine production is diminished, which parallels the reduction in M-cell numbers [21].

#### Mucus layer and AMPs: age-related decline of the biochemical barrier

The gastrointestinal mucosal surface is covered by a layer of mucus, which plays a crucial role in protecting the intestinal epithelium. The mucus layer not only acts as a biochemical barrier to prevent direct contact between IECs and microbes but also shapes the immunological microenvironment and provides an optimal habitat for healthy microbiota. During the aging process, changes in mucus thickness and chemical structure can alter the intestinal environment, which may have significant impacts on the microbial community and intestinal inflammation [27]. In the ileum of aged mice, a slight increase in the number of goblet cells per villus results in larger mucin granules, indicating an increase in mucus abundance [128]. Although the number of goblet cells and mucus in the ileum of aged mice has increased, the number of epithelial-related bacteria has increased, indicating that the protection provided by the mucus layer has decreased [129]. Compared with that in young mice, the thickness of the colonic mucus layer in aged mice is decreased, and supplementation with *Lactobacillus plantarum* WCFS1 can prevent this decline [130]. However, the thickness of the gastric and duodenal mucus layers is not significantly different between young and healthy older individuals [131]. Additionally, protein glycosylation is affected by aging. AMPs interact with the microbiota to maintain gut microbiota stability and immune regulation [132]. The level of important AMPs in the ileum of mice, such as α-defensins and lysozyme produced by Paneth cells, decreases with age [128]. Moreover, aging affects the adhesion of mucus to the gut microbiota, which is considered a precondition for the initial colonization and subsequent proliferation of the gut microbiota. *Bifidobacterium* is an important beneficial intestinal microorganism. *Bifidobacterium*, as a probiotic in the intestine, has many vital physiological functions related to human health, such as acting as a biological barrier, providing nutrition, exerting antitumor effects, enhancing immunity, improving gastrointestinal function, and exerting antiaging effects. Unfortunately, mucosal adhesion to *Bifidobacterium* decreases with age in humans, which explains the reduced abundance of *Bifidobacterium* in the gut microbiota of elderly individuals.

## Gut Immunosenescence and Associated Pathologies

**Figure 3:**
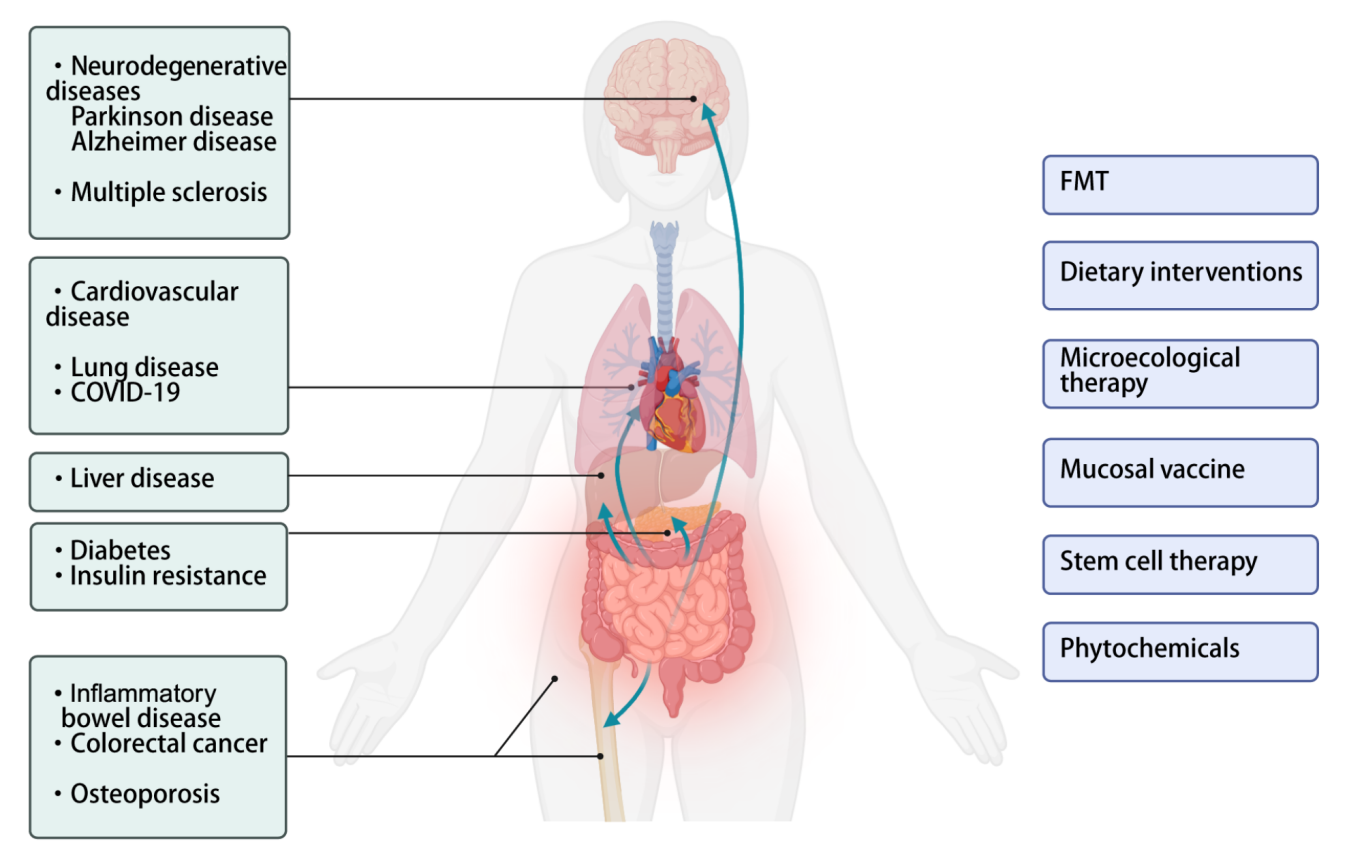
Figure 3 Implications of age-related intestinal dysbiosis for disease and therapy Created in BioRender. An J (2025) https://BioRender.com/33ukol8.

The dysregulation of GALT during aging instigates a cascade of pathologies, prominently featuring increased susceptibility to infections, chronic inflammation and autoimmune disorders ( Figure 3). In advanced age, the gut microbiota not only affects the health of the intestine itself but also regulates the functions of distant organs through the complex “gut-organ axis”. The gut microbiota influences multiple distant organs, including the brain, liver, and lungs, by regulating metabolites, immune cell migration, and neuroregulatory signals. Aging diminishes intestinal barrier integrity through reduced mucus secretion, altered tight junctions, and impaired antigen-specific IgA responses [133]. The compromise of the intestinal barrier is also known as the “leaky gut” [134], which facilitates microbial translocation, triggering systemic inflammaging, a state characterized by elevated proinflammatory cytokines that exacerbate age-related diseases [ 135– 137] . For example, the gut-derived metabolite SCFA regulates osteoclast generation through a Th17/Treg imbalance, while inflammaging suppresses osteoblasts [ 138, 139] .

### Dysbiosis-immunosenescence-inflammaging triad: aprocarcinogenic microenvironment in the gut

In the gastrointestinal tract, dysbiosis-driven depletion of commensals such as *Bifidobacterium* and *Lactobacillus* and overgrowth of pathobionts such as *C*. *difficile* disrupt immune homeostasis, directly contributing to IBD and colorectal cancer (CRC) [ 138, 140, 141] . *Fusobacterium nucleatum* and specific strains of *Bacteroides fragilis* activate β-catenin signaling and promote inflammation, which are associated with the progression of CRC [142]. In carcinogenesis, this triad—immunosenescence, dysbiosis, and inflammaging—creates a permissive microenvironment. Intestinal dysbiosis elevates carcinogenic risks through multiple mechanisms: diminished immunosurveillance of neoplastic cells due to impaired CD8 ^+^ T-cell cytotoxicity and NK-cell activity [ 143– 145] , chronic inflammation-driven DNA damage via reactive oxygen species (ROS) and pro-inflammatory cytokines [146], and direct microbial oncogenesis, as exemplified by *Enterobacteriaceae*-mediated genotoxin production [147]. The immune escape of cancer further exemplifies systemic consequences. Senescent dendritic cells fail to prime cytotoxic T cells, permitting tumor proliferation. Gut dysbiosis reduces CD8 ^+^ T-cell infiltration in tumors and impairs checkpoint inhibitor efficacy, particularly in aging hosts [ 148– 150] .

### The gut-brain axis: microbial dysbiosis and neurodegenerative disease

In addition to the gut, immunosenescence amplifies neuroinflammation via the gut-brain axis, which refers to the cross-talk between the central and enteric nervous systems, which is dominated by signaling from the brain to the gut microbiota and vice versa. Over the past decade, a plethora of evidence has indicated that microbial imbalance and dysfunction of the intestinal epithelial barrier are associated with a variety of neurological disorders. For example, Alzheimer’s disease (AD) patients present a decrease in microbial diversity and significant differences at various classification levels [151]. Butyrate deficiency impairs histone deacetylase 3 (HDAC3)-mediated modulation of tuft cells, dysregulates type 2 immunity and promotes neurotoxic cytokine release ( *e*. *g*., IL-1β and IL-6), which accelerates AD progression [ 152– 154] . Gut leakiness has also been implicated in the pathogenesis of AD and multiple sclerosis (MS) [ 155, 156] . Parkinson’s disease (PD) is a common neurodegenerative disease in elderly individuals who results in a series of motor and nonmotor symptoms. Keshavarzian *et al*. [157] demonstrated differences in the colonic and fecal microbiota in PD patients compared with healthy controls; anti-inflammatory bacteria were less abundant in PD patients, whereas pro-inflammatory bacteria were more prevalent. In the colonic epithelium of PD patients, the expression of occludin is reduced, and the normal distribution of ZO-1 is disrupted [158]. Moreover, the levels of calprotectin and alpha-1-antitrypsin/zonulin, which are markers of intestinal inflammation and permeability, respectively, are elevated in the fecal material of PD patients [159], further indicating that intestinal permeability contributes to the pathogenesis of these neurological disorders.

### Gut-liver axis: barrier breakdown fuels hepatic inflammation and metabolic disease

The gut-liver axis refers to the bidirectional communication between the intestine and the liver. On the one hand, the liver communicates with the intestine by producing bile acids and releasing them into the intestine through the biliary tract. On the other hand, in the intestine, the gut microbiota metabolizes dietary components, bile acids, and other environmental factors, which then enter the liver via the portal vein and regulate metabolic functions. In the case of an impaired intestinal barrier, the translocation of bacteria-derived microbe-associated molecular patterns (MAMPs) to the liver increases. These MAMPs bind to PRRs on liver non-parenchymal cells, leading to the activation of pro-inflammatory and profibrotic signaling cascades [160]. The increased leakage of LPS and other MAMPs from the intestine contributes to the aging of hepatic phenotypes. Supplementation with intestinal alkaline phosphatase in aged mice not only resolves aging-induced intestinal barrier dysfunction but also reduces the age-related increase in liver enzyme levels [161]. In addition, metabolic disorders such as nonalcoholic fatty liver disease (NAFLD) and insulin resistance arise from bile acid dysmetabolism and LPS translocation, which activate hepatic macrophages and stellate cells [ 162– 164] . Type 2 diabetes mellitus (T2DM) associated with advanced age is a chronic and metabolic disease that is caused mainly by insufficient insulin secretion and/or insulin resistance [165]. A cohort study involving 16S rRNA sequencing in northern China revealed that overall intestinal microbiota diversity and the number of butyrate-producing bacteria were significantly lower in diabetic patients than in healthy controls [166].

### Gut-lung axis: respiratory consequences of intestinal dysbiosis

In recent years, the interaction between the intestine and respiratory system has gradually attracted attention, leading to the formation of the concept of the “gut-lung axis”. The latest research revealed that *Tritrichomonas musculis* ( *T*. *mu*), a gut commensal protozoan, drives trafficking of gut-derived ILC2s to the lungs and forms a complex cooperative network with local immune cells, thereby exacerbating asthma and boosting shielding against *Mycobacterium tuberculosis* ( *M*. *tb*) [167]. In elderly individuals, increased bacterial translocation caused by a “leaky gut” can lead to pulmonary dysfunction. Three million people die from chronic obstructive pulmonary disease (COPD) each year, with 92% of these fatalities occurring in people over 60 years of age [21]. Advanced age is a well-established clinical risk factor that may facilitate intestinal dysbiosis and contribute to the development of COPD [ 21, 168] . Increasing dietary fiber can improve COPD-related health. The COVID-19 pandemic further revealed the vulnerability of the gut-lung axis to aging. SARS-CoV-2 infection exacerbates gut dysbiosis, diminishing SCFA-producing bacteria and increasing proteobacterial pathobionts [ 169, 170] . This dysbiosis amplifies systemic inflammation via “cytokine storms”, whereas impaired mucosal immunity compromises viral clearance [ 21, 171] . Long COVID syndrome mirrors accelerated immunosenescence, with persistent T-cell exhaustion and inflammaging perpetuated by unresolved dysbiosis.

### Gut-kidney axis: bidirectional interaction between the gut microbiota and renal function

The gut-kidney axis describes the bidirectional communication and mutual influence between the gut microbiota/metabolites and renal function. Chronic kidney disease (CKD) has a high global prevalence, with gut-kidney axis dysregulation being a key mechanism in its progression. In CKD patients, uremic dysbiosis and intestinal barrier dysfunction exacerbate disease progression [172]. Gut microbiota disruption elevates the production of uremic toxins ( *e.g*., indoxyl sulfate and p-cresyl sulfate), which not only impairs renal function but also promotes systemic inflammation [173]. For example, one study investigated the therapeutic effects of astragaloside IV (AS-IV) on cyclosporine A-induced chronic nephrotoxicity (CICN), elucidating its mechanism through modulation of the “gut-transcriptome-metabolome coexpression network” within the gut-kidney axis framework [174]. Moreover, impaired intestinal barrier integrity, aberrant microbial metabolites, and gut mucosal immune dysregulation play pivotal roles in various renal pathologies [175]. Dietary interventions significantly influence microbiota modulation and CKD progression [176]. PFAS exposure has been associated with renal impairment via gut dysbiosis and altered plasma metabolites, triggering renal inflammation, oxidative stress, and metabolic disturbances that compromise kidney function. Microbiota-targeted therapies ( *e*. *g*., probiotics, prebiotics, or FMT) represent promising therapeutic strategies for kidney diseases [ 175, 177] . In the field of aging research, the gut-kidney axis has emerged as a focal point, with studies on its association with aging continuing to advance. The interactions within this axis are highly relevant during aging, where gut microbiota dysbiosis may contribute to the development of aging-related kidney diseases. Research by teams, including Huashan Hospital, Fudan University, based on population data from the Rugao Longitudinal Aging Study (RLAS), revealed that gut microbiota-mediated ammonia metabolism correlates with age-related renal function decline and the severity of CKD. Circulating ammonia accumulation exacerbates CKD progression, whereas the gut bacterium Segatella copri alleviates ammonia-aggravated CKD through its ammonia-assimilating gene *asnA* [178]. Furthermore, during aging, the gut microbiota-derived metabolite phenylacetylglutamine (PAGln) has a strong age-dependent correlation. Fudan University researchers demonstrated that plasma levels of PAGln and its precursor phenylacetic acid increase with age [81]. The gut microbiota of older adults exhibits enhanced PAA-producing capacity. Critically, PAGln induces cellular senescence *in vitro* and *in vivo* and promotes tissue aging in murine kidneys and lungs. In summary, the gut-kidney axis influences renal function and aging through multiple mechanisms—including microbial imbalance and aberrant metabolite production. Future interventions targeting this axis may offer novel strategies to delay aging and prevent age-related kidney pathologies.

### Gut-mammary axis: effects of gut bacteria and their metabolites on mammary gland health

The gut-mammary axis reveals the potential role of the gut microbiota in mammary gland health, particularly in mastitis. Mastitis is an inflammatory disease that causes significant economic losses in animal husbandry. Although it is commonly attributed to localized inflammatory responses triggered by pathogenic infections of the mammary gland, clinical cases often present with concurrent gastrointestinal disorders [179]. These findings suggest that gut dysbiosis may induce mammary inflammation through a “gastroenterogenic mastitis” mechanism [ 179, 180] . One study revealed that long-term high-concentrate diet feeding induced mastitis in dairy cows, accompanied by an increased abundance of *Stenotrophomonas maltophilia*, a bacterium capable of migrating to the mammary gland and activating the calcium-ROS-AMPK-mTOR-autophagy pathway to promote mastitis [180]. These findings provide novel insights into mastitis etiology, highlighting the role of the gut microbiota in remote organ inflammation and potentially offering new intervention targets for the prevention and treatment of mammary disorders [179]. With aging, the function of the immune system gradually declines, and the stability and diversity of the gut microbiota decrease. This may increase the risk of harmful bacteria translocating to the mammary gland, thereby increasing the probability of mastitis in elderly individuals. Maintaining a healthy gut microbiota may help reduce the risk of mastitis and promote healthy aging of the mammary gland.

### Gut reproductive axis: regulation of reproductive health by the gut microbiota

The gut-reproductive axis refers to the bidirectional interactions between the gut microbiota and reproductive system functions, influencing fertility health in both males and females. In females, the gut microbiota has been recognized as a crucial systemic modulator of reproductive health, significantly impacting endometrial function, embryo implantation, pregnancy maintenance, and parturition timing. Research has elucidated how gut microbial communities influence reproductive biology through molecular signaling pathways, particularly via the gut-endometrium axis [181]. For example, endometriosis, a chronic, estrogen-driven gynecological disorder, may involve gut microbiome-mediated mechanisms through immune modulation, estrogen metabolism, and inflammatory networks [182]. In males, emerging evidence suggests that the gut microbiota may affect long-term fetal and childhood health by influencing the sperm epigenome and DNA methylation patterns [183]. Although the gut microbiota-male infertility connection requires further validation, a proposed hypothesis links environmental pollutants, diet, and the gut-testis axis, suggesting microbial involvement in male reproductive health [184]. As women age, the reproductive system undergoes functional decline, estrogen levels decrease, and corresponding changes in the composition of the gut microbiota occur [185]. These alterations may exacerbate reproductive aging processes, leading to reduced fertility and increased risk of related disorders. In males, the gut microbiota similarly influences testosterone levels and sperm quality, thereby affecting fertility [186]. Preserving the gut microbial balance may help delay reproductive system aging, increase fertility potential, and mitigate associated disease risks.

These studies demonstrate that the gut microbiota engages in complex interactions with other organ systems through multiple axes during the aging process. By modulating the gut microbiota, it may be possible to ameliorate multiple aging-related disorders and delay the aging process. Future research should delve deeper into the molecular mechanisms underlying these axes and develop more effective intervention strategies.

### Microbial drivers of cardiovascular aging

Recent studies have emphasized that intestinal dysbiosis is a key factor in the development of cardiovascular diseases (CVDs) [ 187, 188] . A lower abundance of Bacteroidetes, as well as intestinal dysbiosis involving opportunistic pathogens, are both associated with atherosclerosis [189]. Both rat models and humans have confirmed that hypertension is related to a reduction in the diversity of the intestinal microbiota. SCFAs derived from the intestinal microbiota can help regulate blood pressure through the olfactory receptor Olfr78 and G protein-coupled receptor, while LPS can affect the stability of atherosclerotic plaques [ 190, 191] . However, in the intestines of elderly individuals, the level of SCFAs decreases significantly, and the leakage of LPS increases.

## Therapeutic Interventions Targeting Gut-immune Axis

The intestine is a critical interface between the host and nutrients or the microbiota. Several studies have shown that appropriate measures, such as FMT, dietary interventions, microecological therapy, mucosal vaccine optimization, stem cell therapy and phytochemicals, can help combat intestinal aging ( Figure 3). Furthermore, alterations in intestinal and microbial metabolites resulting from these approaches can provide valuable insights into the underlying mechanisms involved.

### FMT

The connection between aging and the gut microbiota strongly influences human health. Just as the enrichment of certain SCFA-producing bacteria is related to longevity, the decline in overall microbiota richness seems to be a predictor of mortality in the elderly population. To this end, one therapeutic approach under investigation for treating certain diseases, including infections and malignancies, involves FMT. FMT has emerged as a transformative strategy for restoring gut-immune axis integrity in immunosenescence-driven conditions. FMT has been effectively used to treat refractory *Clostridium difficile* infection and shows promise in the treatment of IBD [ 192, 193] . By transferring the donor microbiota, FMT reverses dysbiosis, enhances epithelial barrier function via tight junction proteins and modulates inflammatory pathways. Research has demonstrated that transferring intestinal microbes from healthy mice to germ-free recipients results in substantial increases in body weight, despite the maintenance of standard dietary intake. FMT reverses dysbiosis in elderly IBD patients, reducing endoscopic severity scores in pilot trials [194]. In IBD, FMT achieves clinical remission in ulcerative colitis patients by rebalancing Tregs and reducing pro-inflammatory cytokines [ 195, 196] . However, challenges persist, including variable donor engraftment and risks of pathogen transmission, necessitating standardized protocols.

### Dietary interventions and microecological therapy

Dietary interventions may be the most straightforward approach to buffer age-related dysbiosis. The avoidance of a high-fat diet, processed foods, and red meat, in favor of a high-fiber Mediterranean diet, rich in fruits, vegetables, and whole grains, can promote the growth of beneficial bacteria and increase microbial diversity [ 197, 198] . Caloric restriction leads to changes in the composition of the gut microbiota, which contributes to healthy aging, such as increased levels of *Lactobacillus* [199]. In addition to adjusting the daily dietary status of elderly individuals, microecological therapy can be appropriately utilized to enhance intestinal health and immunity. Probiotics and prebiotics restore microbial diversity, enhancing barrier function and IgA production [200]. MD dysbiosis is correlated with reduced IgA-secreting plasma cells, whereas probiotics such as *Lactobacillus* strains can augment mucosal immunity by promoting IL-10 secretion and dendritic cell maturation, thereby increasing seroconversion rates in preclinical models. Probiotics and synbiotics offer targeted modulation of gut-immune crosstalk, particularly in age-related chronic diseases. *Lactobacillaceae* and *Bifidobacterium* strains enhance gut barrier integrity by increasing mucin production and fortifying tight junctions, while their metabolites ( *e*. *g*., SCFAs) inhibit NF-κB signaling to attenuate systemic inflammation. In AD models, probiotics such as *Lactobacillus plantarum* reduce neuroinflammation by decreasing amyloid-beta plaques and microglial activation through gut-brain axis communication, which is correlated with improved cognitive scores [ 201, 202] . While probiotics have shown health advantages, paraprobiotics are composed of nonliving microbial cells with greater stability and safety and no risk of causing bacteremia or fungemia [203].

Moreover, nutritional interventions have emerged as promising therapeutic strategies for modulating the gut-brain axis and addressing age-related cognitive decline and neurodegenerative diseases. Recent studies have focused on the role of dietary components, such as polyphenols, flavonoids, and specific macronutrients, in influencing the gut microbiota composition and brain health [ 204, 205] . Research has shown that dietary interventions, such as caloric restriction, polyphenol-rich diets, and specific nutrient combinations, can modulate the gut microbiota-gut-brain axis, thereby influencing brain function and cognitive health [206]. For example, studies have demonstrated that dietary flavonoids may ameliorate aging-related cognitive decline by targeting the gut-brain axis [207]. Additionally, nutritional interventions are being explored as a means to delay brain aging and prevent neurodegenerative diseases.

### Mucosal vaccine optimization

Vaccines remain pivotal for preventing age-related infections, yet immunosenescence severely compromises their efficacy. For example, aging reduces vaccine-specific antibody production in some cohorts, which is attributed to T-cell exhaustion and dendritic cell dysfunction [ 208, 209] . To counteract this, novel adjuvant designs leverage toll-like receptor agonists and nanoparticle carriers to increase antigen presentation. Studies have demonstrated that adjuvants such as MF59 improve influenza vaccine responses in older adults by increasing neutralizing antibody titers and T-helper cell activation [210]. Nanoparticle-based oral vaccines targeting PPs increase IgA seroconversion rates in aged models, overcoming dendritic cell dysfunction [ 211, 212] . Importantly, the role of the gut microbiota in vaccine immunogenicity is increasingly recognized. Clinically, probiotic adjuncts in vaccine regimens increase antibody responses; for example, oral administration of jujube powder elevates HSA-specific IgG1 by modulating arginine-metabolizing microbes, highlighting microbiota-directed precision nutrition. Despite their promise, strain-specific effects and dosing variability limit their broad applicability.

### Stem cell therapy

Stem cell therapy, particularly mesenchymal stem cells (MSCs), has immunomodulatory potential in the context of gut immunosenescence. Human amniotic membrane-derived MSCs (hAMSCs) mitigate acute graft-versus-host disease (aGVHD) by reestablishing microbial diversity and enhancing intestinal barrier function [213]. hAMSC transplantation increases *Clostridiales* abundance in murine models, which promotes Treg differentiation via TGF-β secretion, reducing histopathological scores and improving survival rates [214]. These cells also suppress Th1/Th17 polarization and inhibit macrophage-derived IL-23, which is pivotal in autoimmune pathologies. While clinical translation is nascent, early-phase trials have shown reduced colitis severity via IL-22-dependent epithelial repair, although long-term engraftment and safety require further validation.

### Phytochemicals

Future directions include engineered adjuvants that mimic microbial metabolites to train innate immunity, potentially extending durable protection. Phytochemicals such as curcumin and polyphenols exert senolytic effects by suppressing SASP secretion and NLRP3 inflammasome activation. Curcumin downregulates NF-κB signaling, attenuating colonic inflammation in rodent models of aging-related colitis [ 215, 216] . Bile acid agonists ( *e*. *g*., FXR agonists) restore intestinal integrity and mitigate metabolic inflammation, while HDAC inhibitors rejuvenate epithelial HDAC3 function to normalize tuft cell responses [162]. Metformin is the prescribed oral antidiabetic therapy that is suggested to potentially slow the aging process in both animal and human models, with beneficial effects on diabetes, cognitive function, and cancer in humans [ 151, 217, 218] . Metformin can prolong lifespan through alterations in microbial folate metabolism, increase the yield of beneficial microbial products and enhance the intestinal barrier [219], which together help shift the balance away from inflammaging and support healthy aging. Resveratrol (RSV) is a natural non-flavonoid polyphenolic compound that confers intestinal permeability benefits by increasing the expression of tight junction proteins [75].

Collectively, these interventions highlight the gut-immune axis as a therapeutic frontier in aging. Integrating FMT, probiotics, and stem cells with advanced vaccine platforms offers synergistic benefits, such as microbiota-driven Treg induction, which enhances adjuvant efficacy. However, heterogeneity in aging phenotypes demands precision medicine approaches. Ongoing research must address scalability, mechanistic depth, and longitudinal outcomes to translate these strategies into clinical practice.

### Clinical challenges

In the context of clinical application, several therapeutic interventions for aging-related conditions face significant challenges. For example, while FMT shows promise in modulating the gut microbiota and improving health outcomes, it faces challenges related to the standardization of protocols, long-term efficacy, and potential risks of infection or immune reactions [220]. At present, there is a lack of standardized operating procedures and guidelines for optimal selection methods for microbiota preparation and administration in FMT, which increases uncertainty and risks in clinical applications. Although donor screening plays a key role in reducing the risk of infectious diseases, there is still the possibility of pathogen transmission through FMT. In addition, FMT may trigger immune responses, such as mild allergic reactions or more severe immune rejection, in the recipient. Currently, there is still a lack of effective strategies and methods to ensure long-term stable colonization and functional maintenance of the transplanted microbiota. Similarly, dietary interventions and microecological therapy are often hampered by variability in individual responses, difficulty in standardizing dosages, and challenges in ensuring consistent adherence to dietary recommendations [221]. There are differences in metabolic and immune system functions among different individuals. For example, some individuals may have a strong ability to metabolize specific dietary components (such as dietary fiber), enabling them to better utilize these components to regulate the intestinal microbiota and improve health conditions, whereas others may have a weak metabolic capacity and fail to obtain the expected benefits. The standardization of doses for microecological therapies also faces challenges. Probiotics of different strains vary in activity, colonization ability, and metabolic functions, making it difficult to determine a unified effective dose. Mucosal vaccine optimization and stem cell therapy also face hurdles in terms of scalability, safety concerns, and the need for rigorous long-term clinical trials to establish efficacy and safety profiles [222]. Mucosal vaccines may trigger local or systemic immune responses, and in severe cases, they can induce allergic reactions or autoimmune diseases. The ethical issues involved in stem cell therapy should also not be overlooked. In general, these clinical challenges highlight the need for further research and development to optimize these interventions for broader clinical application.

## Conclusion and Remarks

Intestinal immunosenescence represents a critical nexus in organismal aging, orchestrating systemic immune dysfunction through tripartite mechanisms: a cell-autonomous decline in mucosal immune efficacy, dysbiosis-fueled inflammatory cascades, and structural disintegration of the gut barrier. Current evidence unequivocally establishes that aging reshapes innate and adaptive immunity within the gut mucosa, manifesting as attenuated phagocytic capacity of macrophages, skewed T-cell differentiation toward exhausted phenotypes, and diminished secretory IgA production by B cells. These alterations are inextricably linked to microbial dysbiosis characterized by reduced butyrate-producing taxa and pathobiont expansion, creating a self-perpetuating cycle in which microbiota-derived metabolites ( *e*. *g*., LPS and SCFAs) modulate immune cell senescence, whereas barrier leakage amplifies systemic inflammation.

Therapeutically, dietary interventions, microecological therapy and phytochemicals demonstrate promise in restoring microbial ecology and barrier integrity, with preclinical models confirming that *Bifidobacterium* supplementation enhances tight junction protein expression (ZO-1, occludin) while reducing circulating LPS. Similarly, senolytics targeting the SASP axis mitigate epithelial dysfunction and inflammaging in murine models. Nevertheless, critical translational barriers persist: (1) the heterochronicity of immunosenescence across individuals complicates universal therapeutic windows, necessitating biomarkers for staging intestinal immune aging; (2) microbial metabolite-immune receptor interactions ( *e*. *g*., AhR-ILC3 dynamics) remain incompletely mapped, hindering precise microbiome editing; and (3) most interventions fail to address the multidimensional nature of barrier failure-encompassing mucus depletion, junctional disruption, and impaired regenerative capacity of stem cells concurrently.

Emerging technologies herald transformative opportunities. Single-cell multiomics can dissect the spatial architecture of the mucosal immunome, identifying novel targets such as tissue-resident memory T-cell niches or neuroimmune circuits affected by aging. Organoid-based models incorporating aged immune cells and microbiota will accelerate mechanistic validation and drug screening while overcoming the limitations of conventional 2D cultures. Crucially, machine learning frameworks integrating epigenomic clocks ( *e.g*., DNA methylation at barrier-related genes), mucosal metatranscriptomes, and clinical parameters could stratify patients for personalized interventions-rationally combining senotherapeutics, microbiota transplants, and barrier-reinforcing agents such as wogonin or NG-R1—that modulate the TLR4/NF-κB signaling.

Ultimately, conquering intestinal immunosenescence demands interdisciplinary convergence. By elucidating how gut-derived signals accelerate extraintestinal pathologies ( *e.g*., neuroinflammation via the gut-brain axis), we position the gut-immune interface as a master regulator of healthy aging. Future trials must prioritize combinatorial strategies that simultaneously rejuvenate immune competence, restore eubiosis, and regenerate barrier function—only through such integrated approaches—to mitigate the burgeoning burden of age-related diseases rooted in intestinal decline.
